# Periodic Bronchitis in Children

**Published:** 1907-05-25

**Authors:** John Allan


### The General Practitioners' Column.

### PERIODIC BRONCHITIS IN CHILDREN.

By JOHN ALLAN, M.B., Ch.B.Edin.

The ailments of children, many of them of trifling import, form a considerable proportion of the cases which a general practitioner is called upon to treat. What I have termed periodic bronchitis in children is not infrequently met with, and though the condition is not serious, it gives rise to much annoyance to both children and parents, and it requires prolonged and careful treatment. In some instances a specialist may be required to perform an operation, but the onus of carrying out the after-treatment and of bringing about a cure rests entirely with the family medical attendant.

As the names implies, the attacks of bronchitis occur periodically, sometimes at intervals of three or four weeks, perhaps not quite so often. The child feels out of sorts and appears to have a bad cold. There are generally loss of appetite, distaste for food, cough, and constipation. The temperature may or may not be raised, but the rise practically never amounts to more than $\frac{1}{2}^{\circ}$ F. The physical signs in the chest consist merely of some general rhonchi.

As regards etiology, in about 95 per cent. of cases the condition will be found associated with enlarged tonsils and adenoids. A few cases seem to be of the nature of asthmatical attacks, and to treat these is less satisfactory. In many cases rickets is a predisposing cause.

Coming next to treatment, we find that hypertrophied tonsils and adenoid vegetations should be removed; but it must not be thought that this is all that is necessary, for it is only by careful after-treatment that a permanent cure will follow. After removal of enlarged tonsils or adenoids it is important that the child should do respiratory exercises, the aim and purpose of which are to encourage nasal breathing. After, perhaps, years of mouth-breathing the habit will not be an easy matter to check. The following exercises are useful in such cases, and these should be carried out for ten minutes two or three times daily for at least six months after operation. They should not, however, be overdone, and should be stopped before the child tires.

1. Breathe deeply and slowly in and out through the nose, with the mouth close shut.
2. Breathe deeply in and out with the mouth close shut, and one nostril closed by pressing on it with the finger.
3. The same exercises, but with the opposite nostril closed.
4. With arms held above the head, breathe deeply in through the nose with the mouth shut, and out through the mouth with the mouth wide open.

Minute inquiries should be made regarding the child's dietetic régime, as improper feeding greatly aggravates the condition. Sugar and starchy foods should be reduced to a minimum; indeed, it is well to forbid all sweet things. The child should be trained to eat only at meal times, and should not be allowed to have anything in the intervals between the regular meal hours. The diet should consist chiefly of stale bread, dry toast with butter or dripping, bacon, eggs, fish, meat, milk, green vegetables in small quantity, and plain milk puddings (except cornflour, sago, or arrowroot). No tea, coffee, or stimulants should be given. The child should live as much as possible in the open air, the bedroom window should be kept open night and day, and the child should be encouraged to take plenty of exercise. Cold sponging is invaluable; for the first few times it may be as well to employ tepid water, but cold water should be used as soon as possible and will be found most invigorating. Cod-liver oil and iron wine, or an emulsion, may be given, and this is especially valuable if there is, or has been, any rachitic tendency. A change of residence may prove beneficial, and the child should be sent into the country for a time if its home happens to be in a town or city. Cough or other symptoms, if severe, can be treated by appropriate remedies, but under this general tonic treatment which is, I think, the important point in the treatment of such cases, these symptoms will probably improve and finally disappear. In cases of asthmatic nature superficial cauterisation of the septum nasi has been advised, but of this treatment I have had no personal experience.

In conclusion, let me say a few words regarding the prognosis. This can be said to be favourable, for one may say that in all cases a cure will eventually take place. Time is required, and it would be foolish to tell the parents that an immediate cure will result after the removal of tonsils and adenoids. The attacks become less frequent and less severe, and then gradually cease. A few cases resist all treatment, but these, I believe, undergo spontaneous recovery about the age of puberty.

